# Artificial Intelligence Empowered Multispectral Vision Based System for Non-Contact Monitoring of Large Yellow Croaker (*Larimichthys crocea*) Fillets

**DOI:** 10.3390/foods10061161

**Published:** 2021-05-21

**Authors:** Shengnan Wang, Avik Kumar Das, Jie Pang, Peng Liang

**Affiliations:** 1 College of Food Science, Fujian Agriculture and Forestry University, Fuzhou 350002, China; 1180940006@fafu.edu.cn (S.W.); pang3721941@fafu.edu.cn (J.P.); 2 Department of Civil and Environmental Engineering, Hong Kong University of Science and Technology, Hong Kong, China; akdas@connect.ust.hk

**Keywords:** large yellow croaker, freshness, feed-forward neural networks, hyperspectral imaging technology

## Abstract

A non-contact method was proposed to monitor the freshness (based on TVB-N and TBA values) of large yellow croaker fillets (*Larimichthys crocea*) by using a visible and near-infrared hyperspectral imaging system (400–1000 nm). In this work, the quantitative calibration models were built by using feed-forward neural networks (FNN) and partial least squares regression (PLSR). In addition, it was established that using a regression coefficient on the data can be further compressed by selecting optimal wavelengths (35 for TVB-N and 18 for TBA). The results validated that FNN has higher prediction accuracies than PLSR for both cases using full and selected reflectance spectra. Moreover, our FNN based model has showcased excellent performance even with selected reflectance spectra with r_p_ = 0.978, R^2^_p_ = 0.981, and RMSEP = 2.292 for TVB-N, and r_p_ = 0.957, R^2^_p_ = 0.916, and RMSEP = 0.341 for TBA, respectively. This optimal FNN model was then utilized for pixel-wise visualization maps of TVB-N and TBA contents in fillets.

## 1. Introduction

Large yellow croaker (*Larimichthys crocea*), as one of the most economically valuable marine fish in China, has a unique flavor and positive effects on health arising from its constituent proteins, polyunsaturated fatty acids, and carbohydrates, with a predicted substantial market size in south east Asia [1,2,3]. However, due to rigorous environmental requirements and the geographical limitations of large yellow croakers, it is difficult to keep a live large yellow croaker in the market. Low-temperature storage technology is a common technique for preservation and extends the shelf-life of large yellow croaker products due to its convenience and low cost [4]. Protein degradation, lipid oxidation, and microorganism growth are inevitable activities in fish muscle post-mortem due to the high levels of nutrient and moisture content in large yellow croaker, which significantly impact freshness and consumer acceptance and reduce economic efficiency. Therefore, a scalable evaluation system of accurate traits for large yellow croaker freshness is an essential process. 

At present, conventional evaluation methods focus on physical observations, chemical analysis, and microbial activities. Shi et al. investigated the quality changes of mud shrimp during frozen storage by evaluating protein changes and lipid oxidation, and made the prediction using a neural network [5]. Though presenting a comprehensive assessment in the evaluation of fish freshness quality, these traditional methods need high-precision skills and complex manipulations, thus they are time-consuming, inefficient, laborious, and only applicable to small samples. It is difficult to scale them for commercial applications, which would require real-time monitoring of fish’s quality. Therefore, a method involving rapid, non-destruction and efficient testing technology to guarantee quality, safety, and authenticity is desirable for the fish processing industry and consumer market.

Hyperspectral imaging (HSI) technology has been successfully applied in the assessment of fish freshness and safety [6,7,8]. It combines traditional spectroscopy and imaging processing with much higher information in terms of spectral and spatial resolution. It has not only compensated for traditional evaluative methods but also has high-sensitivity, multi-component determination, and intelligent monitoring [9,10]. Cheng et al. have successfully determined the total volatile basic nitrogen (TVB-N) in *Ctenopharyngodon Idella* during frozen storage by HSI technology (400–1000 nm) [11]. Dai et al. have presented a distribution map of texture (hardness, gumminess, and chewiness) of prawn (*Metapenaeus ensis*) using a visible and near-infrared spectroscopy technique [12]. HSI technology was utilized to determine the quality of aquatic products based on some significant features (i.e., color [13], texture [14,15], water content [16], thio-barbituric acid (TBA) [6], and aerobic bacterial count [17]). In addition, several methods were used to develop calibration and prediction models using HSI information, such as partial least squares regression (PLSR), multiple linear regression (MILR), least squares support vector machine (LS-SVM), artificial neural networks (ANN), etc. ANN is usually applied in classification and non-linear regression techniques. Ahmed et al. evaluated the sugar content of potatoes using HSI and FNNs (feed-forward neural networks, radial basis functions neural networks, and exact design redial basis functions) [18]. Peter et al. reported a rapid method to identify pathogenic bacteria using Fourier transform-infrared (FT-IR) HSI and ANN [19]. HSI technology has been widely used in assessing product quality and demonstrated excellent predictability. This is the inspiration for us to explore the possibility of using the combination of HSI technology with FNN for assessment of the freshness of large yellow croaker.

Therefore, this study aims to correct the above imperfections by the following procedures: (1) to develop a HSI system within the spectral region 400–1000 nm to obtain visible and near-infrared (VIS-NIR) hyperspectral images of large yellow croaker under low-temperature treatments (4 °C, 0 °C, and −3 °C); (2) to select optimal wavelengths that are essential to minimize redundant information from HSI and to obtain accurate predictions of TVB-N (an authoritative trait for assessing protein degradation [11]) and TBA (a typical index for accessing lipid oxidation [20]) values, due to the significant difference between fresh and un-fresh samples with large yellow croaker; (3) to develop novel processing algorithms by feed-forward neural networks; (4) to visualize the TVB-N and TBA distribution maps by applying imaging process algorithms for predicting TVB-N and TBA contents of each pixel from the hyperspectral image of large yellow croaker.

## 2. Materials and Methods

### 2.1. Sample Preparation

**Figure 1:**
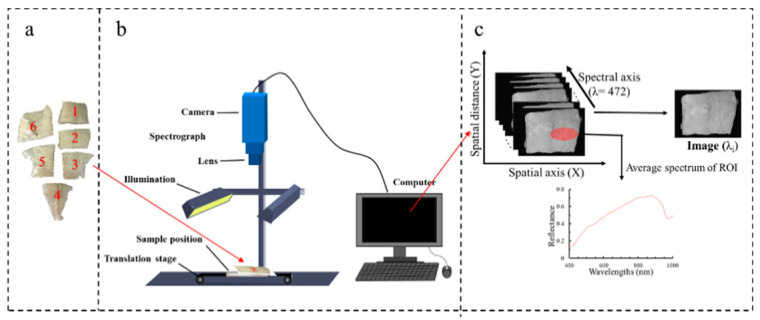
(**a**) Large yellow croaker fillet samples, (**b**) hyperspectral image acquisition, and (**c**) schematic diagram of three-dimensional hyperspectral image.

Fifteen fresh large yellow croakers with an approximate length of 38 cm, were used in this study. These fish were sourced from Zhangwan Dock (Ningde, Fujian Province, China). They were then transported to the laboratory chilled in ice within 2 hours of being caught. Upon arrival, each fish was beheaded, gutted, peeled, and washed with cold water. They were then filleted and each fillet was divided into 5–6 pieces (as shown in Figure 1a). These pieces were further classified based on their relative location on the fish body:dorsal, ventral, and tail. The shape and thickness of the pieces were naturally (slightly) different depending on the body structure of the fish. Finally, the fillets of each fish were packaged in an individual zip-lock bag. These bags were then placed into a refrigerator (Haier, Fuzhou, China). The zip-lock bags were randomly stored at 3 temperatures: −3 °C, 0 °C, and 4 °C. The sampling time for measuring TVB-N and TBA of large yellow croaker fillets were: 0, 4, 8, and 10 days at 4 °C; 4, 8, 12, and 16 days at 0 °C; and 4, 8, 12, 16, 20, and 24 days at −3 °C.

### 2.2. Determination of Quality Indicators

In this study, TVB-N and TBA values were investigated to comprehensively assess the quality changes of large yellow croaker during three different temperature storages. TVB-N as an authoritative parameter for assessing the degradation of protein presents the amount of trimethylamine, dimethylamine, ammonia, and methylamine caused by the decomposition of nitrogenous compounds [21,22]. TBA is considered as a parameter for evaluating the secondary lipid oxidation degree [23]. 

The TVB-N value was measured by a Kjeldahl nitrogen method following standard (GB 5009.228-2016) with modifications. The process is briefly described as follows: 2.00 g of large yellow croaker muscle was weighed and mixed with 28 mL purified water (Milli-Q, Bedford, MA, America) by homogenizing for 60 s. Afterward, the mixture and 0.5 g magnesia were placed in a distilling tube in Kjeltec 8400 instrument (FOSS, Hilleroed, Denmark). Operational parameters were as follows: 30 mL of boric acid (10 g/L), a mixed indicator-bromocresol green (0.1%): methyl red (0.1%) = 10:7; distilling for 3 min; titrated with hydrochloric acid (HCl, 0.01 M).

TBA value was determined as a typical index for accessing lipid oxidation by following the procedure of Salih et al. with some modifications [24]. The process is described as follows: 2.00 g of large yellow croaker muscle was weighed and mixed with 10 mL of purified water. The mixture was homogenized for 60 s and mixed with 10 mL of trichloroacetic acid (TCA, 20%) standing for 20 min, and centrifuging for 5 min at 4200 rpm. The supernatant was at a constant volume to 25 mL with purified water. Then, 5 mL of diluted and 5 mL of TBA (0.01 M) were heated in a 95 °C water bath for 20 min and then cooled down to room temperature. The absorbance of the cooled solution was tested at 532 nm by a spectrophotometer (UV-2601, Beifen-Ruili, Beijing, China) and 1, 1, 3, 3-tetrameth-oxypropane was used to perform a standard curve ranging from 0 to 0.25 ppm. The contents of TBA were expressed as mg/kg sample.

### 2.3. Hyperspectral Imaging System, Images Acquisition, and Processing

A push-broom VIS-NIR HSI system with a wavelength region from 400 to 1000 nm was used to acquire hyperspectral images of large yellow croaker (as shown in Figure 1b). This mainly consists of a charge-coupled device (CCD) camera (DL-604 M, Andor, Ireland) with a high resolution of 1024 × 472 pixels, imaging spectrograph (Isuzu Optics, Taiwan, China), a camera lens (M0814-MP2, Tsukishima, Tokyo, Japan), two 150 W halogen lamp light source (3900e, Illumination Technologies, Taiwan, China) with an angle of 45° to illuminate the moving platform controlled by a stepping motor (IRCP0076-1COMB, Isuzu Optics, Taiwan, China), a black box, and a desktop computer with hyperspectral images’ data acquisition software (Spectral Image Application, Isuzu Optics, Taiwan, China), regulating the exposure time, motor speed, combining mode, wavelength range, and image acquisition. 

In this study, parameters of image acquisition are 32 cm of distance from the lens to the moving platform; 13 mm/sec of the horizontal movement speed of the moving platform; and 0.1 ms of the exposure time of the camera. Each hyperspectral image has 472 wavelengths with an increment of ~1.27 nm.

It should be noted that various imaging parameters (such as illumination or detector sensitivity) could affect the intensity of the HSI, which could be detrimental. In order to reduce the effect of the variations in illumination, detector sensitivity and camera, and physical configuration, the instrument was first adjusted to reflectance mode by using two extra reference images (black and standard white). The corrected hyperspectral image (R_C_) process could be calculated following Equation (1):

R_C_ = (R_0_ − B)/(W − B) (1)

where R_0_, B, and *W* are the raw hyperspectral images, black reference, and white reference, respectively.

Region of interests (ROIs) within hyperspectral images were selected based on the locations where the reference subsamples had been collected for further assessment (as shown in Figure 1c). Finally, mean reflectance spectra from ROIs were presented as spectral data. Multiplicative scatter correction as the spectral pre-processing method was used to eliminate the undesirable scatter effect from the matrix prior to data modeling in this study [11]. We collected a total of 397 and 316 spectrums for TVB-N and TBA, respectively. This work was executed with ENVI 5.1 (ITT Visual Information Solutions, Boulder, CO, USA) software.

### 2.4. Methodology for HSI Processing

In this work, two different methods are utilized, the first being feed-forward neural networks (FNN). In order to benchmark the performance of FNN, a classical method was also utilized to analyze the same dataset. It was found, that among researchers working with HSI for fish, partial least squares regression (PLSR) method is popular [25,26,27]. Therefore, in this work, PLSR was selected. The following section describes the methods in detail. For this work, we have used Matlab R2020a (The Mathworks, Inc., Mass, Natick, MA, USA) and Unscrambler V9.7 (CAMO, Trondheim, Norway) software for implementation of the following methods. 

#### 2.4.1. Feed-Forward Neural Networks

Feed-forward neural networks (FNN) are one of the most common types of neural network in which the information moves forward. A feed-forward neural network is a state of the art method for solving regression problems such as ours [28]. A FNN consists of three components-layer(s) (of neurons), a linear weighing function (of neurons), and a non-linear activation function (to select the relevant useful neurons). The non-linear function used here is-LeakyRelu. For a given dataset, we have attempted to use FNN with different widths and depths. Then, the optimal number of latent variables depends on the prediction error, typically performed by using the lowest value of the predicted residual error sum of squares [29]. 

#### 2.4.2. Partial Least Squares Regression

Partial least squares regression (PLSR) as a classical method was widely applied to multivariate data analysis, which is regarded as a standard calibration technology due to considering the relation between sample characteristics and spectroscopic data [30,31]. It has performed outstandingly when the wavelength numbers are greater than samples and when there is multicollinearity among variables. The general equation describing the PLSR model, the quantitative relation between independent wavelengths (X) and observations of TBA/TVB-N (Y) results can be described as follows (2) [16]:

Y_n__×1_ = X_n__×1_ × B_k__×1_ + E_n__×1_ (2)

where k is the number of wavelengths for n number of calibration samples, B is the matrix of regression coefficients, and E is the regression residual matrix. 

#### 2.4.3. Selection of Optimal Wavelengths

As explained earlier, data collection involves spectral results at 472 wavelengths; however, the chemical results such as TVB-N and TBA are single-dimensional data. The spectral information extracted from hyperspectral images not only encompasses abundant valid information but redundant information also exits, which needs to be inverted in order to gather the relevant chemical properties. This could easily bottleneck the computational resources in a practical situation leading to higher inference time due to the lower speed of the computational process. One possible way to avoid this is to select effective wavelengths carrying the most valuable information that inflected the alteration of TVB-N and TBA values during multivariate analysis and removing the useless wavelengths with irrelevant information. To do so, the regression coefficient for each wavelength in the spectral content for TVB-N and TBA was first computed. It is well established in mathematics that columns (wavelengths) with lower regression coefficients do not contain as much relevant information compared to those with a higher coefficient [32]. Therefore, we have selected wavelengths corresponding to local maxima (and minima) of regression coefficients. Expectedly, this substantially shrinks the dimensionality of the spectral dataset. Based on these optimal wavelengths, a new simplified FNN-simplified model was established. Again, to benchmark its performance, a simplified version of the PLSR was also developed from optimal wavelengths.

#### 2.4.4. Evaluation of Models

Reflectance spectra acquired from fillets were randomly divided into calibration and prediction datasets, using a hold-out method (85:15 ratio). The abilities of the calibration and prediction performance were evaluated based on six parameters: the correlation coefficients of calibration (r_c_), the coefficient of determination of calibration (R^2^_c_), the root mean square error of calibration (RMSEC), the correlation coefficients of prediction (r_p_), the coefficient of determination of prediction (R^2^_p_), and the root mean square error of prediction (RMSEP). Generally, a satisfactory model should present higher r_c_, r_p_, R^2^_c_, R^2^_p_, and lower RMSEC and RMSEP values.

#### 2.4.5. Visualization of TVB-N and TBA Contents

In order to visually observe the contents of TVB-N and TBA of large yellow croaker fillets during storage, it is necessary to visualize the quantitative spatial distribution by grading color maps for evaluating the corruption of aquatic products. In this work, according to the evaluation of the models, the best model was used to predict the quality indicators’ content at each pixel in the hyperspectral image of the prediction dataset. A distribution map of the chemical indicator within a fillet is based on the spatial position at each pixel and the corresponding indicator’s value.

## 3. Results and Discussion

### 3.1. Statistics of TVB-N and TBA Contents and Spectra

A robust and developed model usually needs a wide range of quality variation for calibration and prediction samples [33]. Table 1 presents the relevant statistics of TVB-N and TBA contents of the examined large yellow croaker fillets. The presence of a wide range of TVB-N and TBA indicators indicated that the calibration dataset had an excellent performance in establishing a reliable calibration model. We used normalized data in the calibration dataset and prediction dataset, which ensures a consistent coverage between the calibration model and prediction model. This not only helps the establishment of stable calibration models but also increases the credibility of the prediction model.

**Figure 2:**
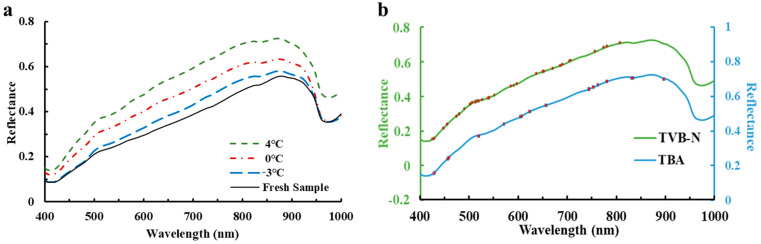
(**a**) Average spectral curves of the examined large yellow croaker fillet after 8 days’ storage; (**b**) Distribution of optimal wavelengths in full reflectance spectra.

The differences in the average reflectance extracted from the pixels of ROIs of the examined large yellow croaker fillets after 8 days’ storage under three different treatments in the spectral range 400–1000 nm are shown in Figure 2a. It is apparently observed that the spectral features showed an analogous changing trend with some broadband adsorption peaks in the whole wavelength region. However, there were still some variations in the magnitude of spectral reflectance, which may be due to the different chemical transformations (i.e., lipid oxidation and protein degradation, and microbial activities) during different temperature treatments, which is consistent with the previous literature [34,35]. In the visible spectral range (400–780 nm), there are two adsorption broadbands located at around 417 nm and 553 nm, which may be due to adsorption of pigments (i.e., ferroheme [36]; hemoglobin and myoglobin [37]; and astaxanthin and canthazanthin [38]). In the NIR spectral range (780–1000 nm), there are two weak adsorption bands at 836 nm (associated with the third overtone O-H stretching [25] and lipid oxidation [39]) and 974 nm (related to the second overtone O-H stretching in water [13]).

### 3.2. Prediction of TVB-N and TBA Contents Using Full Reflectance Spectra

Throughout the average reflectance spectra extracted from hyperspectral images of large yellow croaker fillets and their corresponding indicator (TVB-N and TBA, respectively) values, the prediction models were developed by using PLSR and FNN algorithms within the full reflectance spectra, respectively. Table 2 shows the quantitative relationships of measured and predicted values of TVB-N and TBA of fillet samples. As shown in Table 2, for the TVB-N analysis, PLSR exhibits excellent performance in building the calibration model with the two coefficients of determinations (R^2^_c_ and R^2^_p_) were 0.901 and 0.894, respectively and the two corresponding root mean square errors (RESEC and RESMP) were 5.708 and 6.904, respectively. This shows better performance than another study reported by Liu et al. for the rapid prediction of pH values in salted meat using Vis-NIR HSI technology, showing coefficients of determination (R^2^_c_ and R^2^_p_) of 0.856 and 0.797 using the same PLSR modeling approach [40]. In addition, compared with the PLSR model, the FNN algorithm displayed better effectiveness and predictability with a major increase of 0.081 and 0.091 for R^2^_c_ and R^2^_p_ and a decrease of 3.285 and 4.291 for RMSEC and RMSEP. It is clear that a higher R^2^_c_ and R^2^_p_ and lower RESEC and RESMP are significant in spectral analysis and quantitative prediction. Therefore, the model established by the FNN algorithm using full reflectance spectra has superior prediction accuracy than the PLSR approach for the prediction of TVB-N content of large yellow croaker fillets. Moreover, the FNN approach also exhibited high ability in the prediction of TBA content of large yellow croaker fillets with higher R^2^_c_ (0.945) and R^2^_p_ (0.929) and much lower RMSEC (0.130) and RMSEP (0.133) than that of the PLSR model. More importantly, the capability of the FNN model confirms the efficiency and robustness of HSI technology for TVB-N and TBA content prediction in a rapid and non-destructive technology.

### 3.3. Prediction of TVB-N and TBA Contents Using Selected Spectra

**Figure 3:**
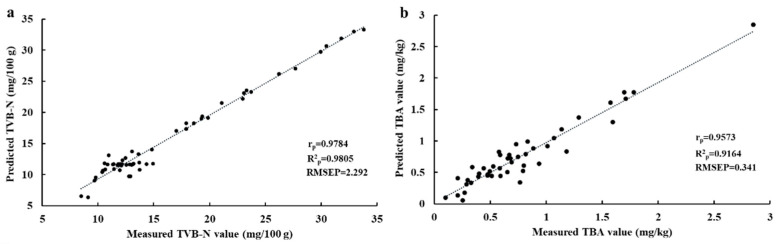
Scatter plots of predicted versus measured TVB-N (**a**) and TBA (**b**) obtained by FNN-simplified model based on the optimal wavelengths.

Optimal wavelength selection is a significant step in eliminating the redundant information of hyperspectral images, optimizing the calibration models, reducing the computation time, and further satisfying the practical application [41]. In this work, the regression coefficient was conducted to select the optimal wavelengths carrying the most valuable information related to large yellow croaker quality from the full reflectance spectra, to simplify the original calibration models. As shown in Figure 2b, 35 individual variables (428, 430, 450, 457, 476, 481, 492, 505, 506, 510, 511, 515, 516, 517, 521, 522, 528, 541, 543, 553, 587, 594, 600, 639, 653, 656, 673, 685, 692, 707, 710, 759, 777, 784, and 811 nm) were selected as the optimal wavelengths to replace the full reflectance spectra for the following prediction of TVB-N content in large yellow croaker fillets, and 18 optimal wavelengths (430, 431, 458, 461, 522, 573, 608, 610, 627, 660, 668, 747, 756, 766, 784, 836, 837, and 901 nm) were obtained for further prediction of TBA values. Notably, most of the selected wavelengths were located in the limit of the visible spectrum (400–780 nm), which is in accordance with the TVB-N content of grass carp reported by Cheng et al. [11]. Table 2 shows the performance of simplifying models of PLSR-simplified and FNN-simplified models established by using the reduced spectral information for TVB-N and TBA indicators, respectively. It can be noticed that although the number of wavelengths was reduced by more than 92% (472 vs. 35 and 472 vs. 18), the prediction ability of optimized models (PLSR-simplified and FNN-simplified) show minor differences compared with the models developed using full spectral regions. As illustrated in Table 2, even in this case comparison with the PLSR-simplified, FNN-simplified model showed superior effectiveness and accuracy in calibrating and predicting TVB-N and TBA values with the R^2^_c_ of 0.978 and 0.930, R^2^_p_ of 0.981 and 0.916, RMSEC of 3.933 and 0.148, and RMSEP of 2.292 and 0.341, respectively, confirming that FNN-simplified is regarded as the best model using optimal reflectance spectra to predict the TVB-N and TBA values for the evaluation of freshness of larger yellow croaker fillets. Figure 3 showed the scatter plot of predicted versus measured TVB-N and TBA obtained by the FNN-simplified model based on the selected wavelengths. It is thus indicated that HSI technology with the selected wavelengths is also suitable for quantitative prediction of TVB-N and TBA values of large yellow croaker filets, respectively.

### 3.4. Distribution Map of TVB-N and TBA Contents

**Figure 4:**
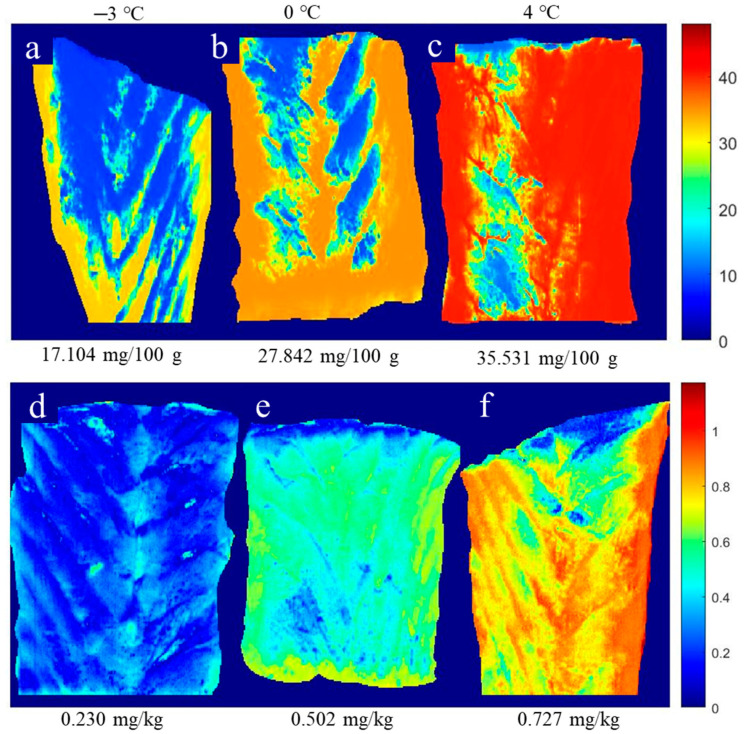
Distribution maps of TVB-N (**a**–**c**) and TBA (**d**–**f**) contents in large yellow croaker fillets after 8-day storage at three low-temperature treatments.

The great advantage of HSI technology is that the spectral and spatial information at each pixel in a hyperspectral image make it possible to reveal the freshness of fish by showing chemical images of the quality indicators obtained by using suitable calibration models [33]. To do so, the FNN-simplified method, obtained with the help of the selected reflectance spectra, was used to transfer information from each pixel in the hyperspectral image to the prediction of chemical values (TVB-N and TBA) in the fillet sample of large yellow croaker. This is because a simplified model is expected to be more representative of a practical scenario. It is expected that pixels with similar characteristics would present similar visualization with similar quality parameters [42]. Figure 4 shows the distribution map of TVB-N and TBA content in large yellow croaker fillets after 8-day storage at three low-temperature treatments (−3 °C, 0 °C, and 4 °C). Differences in TVB-N and TBA contents were observed using images in a fillet, which could imply the degradation of nitrogen-containing chemical compounds (i.e., protein) and the oxidation degree of lipids after 8-day storage at three low-temperature treatments. Figure 4a,b,d,e show that surrounding locations present higher values of TVB-N and TBA than the inner location, which means that the degradation and oxidation degree started at the edge of the fillet. This might be due to the ruptured cells, oxygen penetration, and microorganism activities at the cut surface [6,11]. Moreover, Figure 4c,f show a large area of red color, indicating that a higher level of degradation and oxidation degree has occurred during higher storage temperature (4 °C) than others (−3 °C and 0 °C). Thus, this further illustrates the alternation process of TVB-N and TBA contents in the fillet of large yellow croaker under the different storage treatments by comparing the different distribution maps of TVB-N and TBA contents, respectively. Additionally, it is a successful technique to evaluate the fillets’ freshness by showing the TVB-N and TBA contents via RGB images.

## 4. Conclusions

A VIS-NIR hyperspectral imaging system empowered with PLSR and FNN was conducted to rapidly and non-invasively monitor the freshness of large yellow croaker fillets. The results validated that FNN has showcased excellent prediction accuracies in full and selected reflectance spectra. In addition, the optimal FNN-simplified model was utilized for pixel-wise visualization maps of TVB-N and TBA contents in fillets, revealing the distribution of freshness of large yellow croaker fillets during storage. However, some obstacles need to be overcome, such as the limited number of regression algorithms available, different locations of the fish, and different storage methods. Further experimental validation of this method i.e., combining HSI and FNN, is still needed for its application in the field of aquatic food processing.

## Figures and Tables

**Table 1:** Reference results of total volatile basic nitrogen (TVB-N, mg/100 g) and thio-barbituric acid (TBA, mg/kg) contents of large yellow croaker fillets measured by conventional methods.

Quality Indicators	No. of Samples	Max	Min	Mean ± SD ^1^	Range
TVB-N	397	34.920	8.176	14.518 ± 5.509	26.744
TBA	316	3.072	0.097	0.61 ± 0.48	2.975

^1^ SD: standard deviation.

**Table 2:** Calibration and prediction results of TVB-N and TBA values for large yellow croaker fillet by HSI technology.

Quality Indicators	Model	No. W ^1^	No. LV ^2^	Calibration			Prediction		
				r_c_	R^2^_c_	RMSEC	r_p_	R^2^_p_	RMSEP
TVB-N	PLSR	472	13	0.949	0.901	5.708	0.932	0.894	6.904
	FNN	472	22	0.991	0.982	2.423	0.993	0.985	2.613
	PLSR-simplified	35	10	0.933	0.871	6.510	0.927	0.875	7.668
	FNN-simplified	35	6	0.989	0.978	2.933	0.978	0.981	2.292
TBA	PLSR	472	10	0.934	0.891	0.421	0.922	0.896	0.529
	FNN	472	22	0.972	0.945	0.130	0.964	0.929	0.133
	PLSR-simplified	18	8	0.917	0.860	0.313	0.908	0.887	0.429
	FNN-simplified	18	22	0.964	0.930	0.148	0.957	0.916	0.341

^1^ No. W: number of wavelengths; ^2^ No. LV: number of latent variables.

## Data Availability

The data presented in this study are available on request from the corresponding author.
